# Does Genetic Diversity Predict Health in Humans?

**DOI:** 10.1371/journal.pone.0006391

**Published:** 2009-07-27

**Authors:** Hanne C. Lie, Leigh W. Simmons, Gillian Rhodes

**Affiliations:** 1 School of Psychology, University of Western Australia, Crawley, Western Australia, Australia; 2 Centre for Evolutionary Biology, School of Animal Biology, University of Western Australia, Crawley, Western Australia, Australia; University of Liverpool, United Kingdom

## Abstract

Genetic diversity, especially at genes important for immune functioning within the Major Histocompatibility Complex (MHC), has been associated with fitness-related traits, including disease resistance, in many species. Recently, genetic diversity has been associated with mate preferences in humans. Here we asked whether these preferences are adaptive in terms of obtaining healthier mates. We investigated whether genetic diversity (heterozygosity and standardized mean *d^2^*) at MHC and nonMHC microsatellite loci, predicted health in 153 individuals. Individuals with greater allelic diversity (*d^2^*) at nonMHC loci and at one MHC locus, linked to HLA-DRB1, reported fewer symptoms over a four-month period than individuals with lower *d^2^*. In contrast, there were no associations between MHC or nonMHC heterozygosity and health. NonMHC-*d^2^* has previously been found to predict male preferences for female faces. Thus, the current findings suggest that nonMHC diversity may play a role in both natural and sexual selection acting on human populations.

## Introduction

Positive associations between individual genetic diversity (heterozygosity) and fitness-related traits are reported across many taxa, although the effects are often weak, and the underlying mechanisms are not well understood [1], [2]. Coltman et al. [3] proposed that genetic diversity could influence fitness through disease resistance. Indeed, heterozygosity has sometimes been associated with enhanced disease resistance in nonhuman animals [3]–[7], [but see 8]. Surprisingly little is known about the effect of genome-wide heterozygosity on resistance to disease in humans, but recently consanguinity, resulting in reduced heterozygosity, was associated with increased susceptibility to two severe infectious diseases, tuberculosis and hepatitis [9]. Reduced genome-wide heterozygosity has also been associated with greater incidence of non-infectious diseases such as high blood pressure, high cholesterol levels, stroke, and asthma [10], [11]. Thus, there is some evidence that heterozygosity is beneficial for human health.

Heterozygosity at genes within the MHC (or human leukocyte antigen, HLA, in humans) may be especially important for enhanced immune functioning, and therefore health [12]–[14]. MHC alleles encode peptides that bind to and present a restricted range of foreign antigen-peptides to T-cells, thereby initiating a rapid immune response [15]. Because MHC alleles are expressed codominantly, MHC heterozygotes should be able to detect a broader range of pathogens than homozygotes [12], [16], [17].

Despite being strongly predicted by theory, evidence for a MHC heterozygote advantage is equivocal [e.g. 14], [18], [19]. A MHC heterozygote advantage has been found in non-human animals for multiple infections [20]–[25], and in humans, for resistance to specific complex viral infections and parasites [26]–[29], [but see 30]. In addition, some studies report an advantage for an intermediate level of MHC diversity in non-mammalian species [31]–[33], whereas other studies report no heterozygote advantage, but MHC allele-specific resistance against specific pathogens [34]–[38]. These mixed findings might reflect experimental design rather than a lack of heterozygote advantage *per se*, because resistance is allele specific and many studies test resistance towards only one or a few pathogens [18]. Thus, heterozygote advantage may only be apparent in the context of infections by multiple pathogens [18], [25].

As well as influencing individual fitness, heterozygosity, both within the MHC and in general, appears to play a role in sexual selection [2], [39]. Provided heterozygosity is associated with enhanced fitness, preferences for heterozygosity in a mate should be adaptive. If heterozygous individuals are healthy, then a heterozygous mate could potentially enhance offspring viability directly, through reduced risk of contagion or better provisioning of resources and parental care [40], and indirectly, because heterozygosity is on average heritable [41], [42].

There is evidence that heterozygosity, especially at the MHC, is preferred in a mate [reviewed in 2], [39]. For example, female fur seals (*Arctocephalus gazella*) actively choose more heterozygous mates [42]. Female fat-tailed lemurs (*Cheirogaleus medius*) prefer genome-wide and MHC heterozygosity in their mates [43], and MHC heterozygosity is the best predictor of reproductive success in macaques (*Macaca mulatta*) [44]. Recent studies in humans have found that females prefer the faces [45], [46], [but see 47] and the axillary odour [47] of MHC-heterozygous males. Moreover, males preferred the faces of females with greater general genetic diversity, measured as standardized mean *d^2^* (reflecting genetic distance between parental genomes) at loci outside the MHC [46, H. Lie, L. W. Simmons, & G. Rhodes, unpublished data].

An association between genetic diversity and health would provide evidence that preferences for genetic diversity in a potential mate might be adaptive. Here, we test whether genetic diversity within and outside the MHC is associated with “everyday” health, measured broadly as symptoms of common infectious diseases. Number of self-reported symptoms has been validated as a proxy measure of presence of infectious diseases [48], and has been used previously [e.g. 49], [50], [51]. Susceptibility to infectious diseases has commonly been measured by retrospective, self-report questionnaires, often requiring recall of illness episodes or symptoms that may have occurred in the last year, three years, or even over an individual's lifetime [e.g. 30], [52], [53]. Because recalling illness events over such long periods is subject to memory and reporting biases, we use a longitudinal design measuring health over a four-month period to reduce the influence of such biases on health reporting. Moreover, because health differences between more and less heterozygous individuals could be difficult to detect in a sample of university students in a modern society, the four-month health survey included a stressful exam period to potentially enhance individual differences in susceptibility to common infectious diseases. Elevated stress levels, associated with stressful events such as exam periods, are well known to compromise immune functioning, and increase rates of infectious diseases in students [54], [55].

To examine whether genetic diversity in general and/or at the MHC is associated with health, we used microsatellite markers at loci within and outside the MHC. We calculated two measures of individual genetic diversity: multilocus heterozygosity (referred to as *H*) and standardized mean *d^2^* (referred to as *d^2^*), which reflect the proportion of heterozygous loci and the average genetic distance between alleles within an individual respectively. Mean *d^2^* has been criticised for being less sensitive to genetic diversity-fitness correlations than heterozygosity [e.g. 56], and conditions where mean *d^2^* is expected to outperform heterozygosity are poorly defined [56]–[58]. However, some studies have found mean *d^2^* to predict different aspects of individual fitness than heterozygosity [e.g. 7], [59], [60], or to predict fitness-related traits when there was no effect of heterozygosity [60]–[63]. We therefore included both measures of genetic diversity.

Microsatellites are assumed not to be subject to selection. However, microsatellites embedded within the MHC are increasingly being used to infer levels of MHC diversity, as they typically show evidence of selection acting on the MHC loci to which they are linked [e.g. 64], [65], [66]. The MHC microsatellites used here are known to be in linkage with MHC Class I, II, and III genes [67]. In addition, these microsatellites span a relatively large section of the MHC compared to the 2–5 MHC loci typically sampled in past research [27], [28], [30]. The nonMHC loci were chosen to be qualitatively similar to the MHC loci. Because the nonMHC loci were highly polymorphic, they are likely to be in linkage with nearby functional loci under balancing selection [68], [69]. Thus, our measures of microsatellite diversity should reflect genetic diversity at functional loci across the MHC, and in general. 

In summary, we investigated whether individuals with greater genetic diversity (*H* and *d^2^*) in general and/or at the MHC report better “everyday” health over a four-month period than less genetically diverse individuals. We previously found that genetic diversity predicted mate preferences in the same sample. If genetic diversity predicts health, then this would suggest that the observed preference for genetically diverse individuals is adaptive.

## Methods

### Ethics statement

Procedures were approved by the university's Human Research Ethics Committee (project number RA/4/1/1292), and each participant provided written informed consent.

### Participants

The sample consisted of 153 white Caucasian students at the University of Western Australia (77 females, mean age 19.5, SD 2.5; 76 males, mean age 20.4, SD 3.2, for details see Lie et al. 2008). Each participant provided written informed consent, a DNA-sample, and completed several questionnaires in return for course credit and/or 10 Australian dollars.

### DNA samples and genetic diversity measures

The procedures for DNA collection and genetic analyses are described in full elsewhere (Lie et al. 2008). Briefly, MHC diversity was measured using 12 microsatellite markers (average number of alleles: 12, and heterozygosity: 0.83) across the MHC region, all of which are in linkage disequilibrium with one or more MHC genes [67]. General genetic diversity (nonMHC) was measured using 11 microsatellite markers (average number of alleles: 14, and heterozygosity: 0.84) located on 11 different chromosomes. All loci were in Hardy-Weinberg equilibrium (Lie et al. 2008). Genetic diversity was measured using Heterozygosity (*H*), calculated as the proportion of heterozygous loci within an individual, and a standardized version of mean *d^2^* [59]. Here, *d^2^* is the squared difference in number of repeat units between the two alleles at a given locus, standardized by the maximum observed value at that locus, and averaged across all measured loci [see 46], [63], [70]. We used the standardized version of mean *d^2^* to reduce undue influence of loci with large allelic size ranges on the arithmetic mean of the measure [70]. The resulting values range from zero to one.

Provided microsatellites evolve under the stepwise mutation model [71], [72], mean *d^2^* should reflect genetic distance between parental genomes. Greater *d^2^* values indicate longer time since coalescence and higher levels of outbreeding, while greater values of heterozygosity should better reflect lower levels of inbreeding [73].

### Health measure

Health was measured broadly as the number of symptoms of infectious diseases reported prospectively over a four-month period. Every fortnight, participants completed an online symptom checklist where they indicated which, if any, symptoms they had experienced that fortnight. Symptom included: sore throat, cough, runny or stuffy nose, fever, sinus pain, ear pain, itchy, irritated or runny eyes, vomiting. The participants were asked not to report symptoms due to allergies such as hay-fever. The number of symptoms reported each fortnight was summed and averaged across number of surveys completed for each participant (134 participants completed all eight, 14 completed seven, three completed six, and two participants completed five). Thus a higher score on the health measure reflects a higher average number of symptoms (worse health) reported each fortnight.

A range of potentially confounding variables that can influence susceptibility to infectious diseases was also measured by questionnaires, fortnightly over the four-month period. Stress has a negative impact on immune functioning [54], and was measured using the stress-subscale from the Depression, Anxiety and Stress Scales [DASS 21; 74]. A higher score indicates more stress (range 0–42). Negative affect (NA), a personality trait capturing individual differences in experiencing negative moods, has been found to significantly bias self-report of symptoms [75], and was measured using Stokes and Levin's [76] Negative Affect scale. A higher score indicates higher levels of NA (range 0–147). Participants also reported any of their behaviours that they felt had compromised their health (e.g. excessive drinking, smoking, taking drugs, lack of sleep). Because exercise can affect immune functioning [77], we calculated average amount of self-reported exercise per fortnight (hrs, mins) per participant. Additionally, Socio-Economic Status (SES), commonly associated with health [78], was measured by scoring both parents occupation according to the Australian Standard Classification of Occupations (ASCO, Australian Bureau of Statistics, 2^nd^ edition, 1997) [79]. The lowest score (indicating higher SES) obtained from either parent was used as the SES measure. Lastly, age was included as a potential covariate, because age influences immunity [80].

## Results

The health measure (average number of symptoms) was square-root transformed to achieve normality. All other distributions were skewed, but left untransformed as some could not be transformed to normality. Descriptive statistics for all variables are presented in Table 1, and Pearson's product-moment and Spearman rho correlation coefficients are presented in Table S1 of the online supporting information.

**Table 1:** Descriptive statistics for the health (number of symptoms), covariate and the genetic diversity variables (n = 153).

Variable	Mean (SD)	Median (Range)
Health*	1.07 (0.43)	1.06 (0.00–2.12)
Age	19.92 (2.91)	19.00 (18.00–33.00)
SES	2.62 (1.51)	2.00 (1.00–8.00)
NA*	69.12 (19.37)	68.57 (30.68–115.14)
Stress*	10.64 (6.56)	9.50 (0.00–29.43)
Non-Healthy Behaviour*	0.41 (0.37)	0.29 (0.00–1.14)
nonMHC-*H*	0.84 (0.12)	0.82 (0.55–1.00)
MHC-*H*	0.84 (0.12)	0.83 (0.50–1.00)
nonMHC-*d^2^*	0.16 (0.07)	0.16 (0.04–0.40)
MHC- *d^2^*	0.17 (0.07)	0.17 (0.05–0.35)

* Measures are averages from data collected once a fortnight for a four-month period.

The correlations in Table S1 indicated that the nonMHC-*H* and -*d^2^* measures and the MHC-*H* and -*d^2^* measures were significantly, positively correlated (all *r*>0.3). Moreover, the health measure was associated with several of the covariates. We therefore fitted initial multiple regression models by simultaneously entering all potential covariates as well as a gender term, nonMHC and MHC diversity, and interaction terms between the gender and diversity measures (Table S2 and S3 of the online supporting information). Although the predictor variables were not normally distributed, the transformed outcome variable, health, was. To ensure that the data was suitable for regression analysis, we checked the normality of residuals and for the presence of outliers with high leverage values in the final models. The models were simplified by sequential deletion of non-significant variables. Significant covariates retained in the final models were age, stress and non-healthy behaviours. Increased age predicted fewer symptoms, while increased stress levels and non-healthy behaviours predicted more symptoms reported. No other covariates or interaction terms were significant. Because gender did not interact with genetic diversity, male and female data were combined for further analyses.

With the significant covariates established, we then used hierarchical multiple regression models to investigate whether genetic diversity predicted health (number of symptoms) after controlling for the covariates. Hierarchical regression allows us to examine whether genetic diversity (*H* and *d^2^*) has an effect on health over and above the effect of the covariates by entering the covariates in the first block and the genetic diversity variables in subsequent blocks. Additionally, we entered nonMHC diversity in the second block and MHC diversity in the third block to examine whether MHC diversity influenced health when controlling for nonMHC diversity.

We found no relationship between either nonMHC-*H* or MHC-*H* and health after adjusting for covariates (Table 2). There was, however, a small, but significant, effect of nonMHC-*d^2^* on health, with individuals with greater nonMHC-*d^2^* reporting fewer symptoms (better health) over the four-month period (Table 3). In addition, there was also a small independent effect of MHC-*d^2^* on health after controlling for the effect of nonMHC-*d^2^*, with individuals with greater MHC-*d^2^* reporting fewer symptoms.

**Table 2:** Hierarchical multiple regression model predicting health (number of symptoms) using nonMHC and MHC heterozygosity (H) measures, while controlling for covariates.

Blocks	B (±95 CIs)	SE	*β*	*t*	*p*	*R^2^*	*ΔR^2^*
Block 1							
Age	−0.03 (−0.05–−0.01)	0.01	−0.21	−2.73	0.007	0.15***	
Stress	0.02 (0.01–0.03)	0.01	0.25	3.33	0.001		
Non-healthy behaviour	0.21 (0.04–0.39)	0.09	0.18	2.42	0.017		
Block 2							
Age	−0.03 (−0.06–−0.01)	0.01	−0.21	−2.66	0.009	0.15***	0.00
Stress	0.02 (0.01–0.03)	0.01	0.26	3.33	0.001		
Non-healthy behaviour	0.22 (0.04–0.39)	0.09	0.19	2.42	0.017		
nonMHC-*H*	0.08 (−0.47–0.62)	0.28	0.02	0.28	0.779		
Block 3							
Age	−0.03 (0.06–−0.01)	0.01	−0.22	−2.83	0.005	0.16***	0.01
Stress	0.02 (0.01–0.03)	0.01	0.26	3.36	0.001		
Non-healthy behaviour	0.21 (0.04–0.039)	0.09	0.18	2.37	0.019		
nonMHC-*H*	0.02 (−0.53–0.57)	0.28	0.01	0.08	0.936		
MHC-*H*	0.41 (−0.12–0.93)	0.27	0.12	1.52	0.130		

Covariates were entered in block 1 to investigate whether genetic diversity had an effect on health over and above the effect of the covariates. NonMHC-H was entered in block 2 to examine whether MHC-H had an effect on health over and above the effect of nonMHC-H (n = 153).

Notes: Overall model: *F_5,147_* = 5.56, *p*<0.001.

***p<0.001.

**Table 3:** Hierarchical multiple regression model predicting health (number of symptoms) using nonMHC and MHC standardized mean d^2^ (d^2^) measures, while controlling for covariates.

Block with predictors	B (±95 CIs)	SE	*β*	*t*	*p*	*R^2^*	*ΔR^2^*
Block 1							
Age	−0.03 (−0.05–−0.01)	0.01	−0.21	−2.73	0.007	0.15**	
Stress	0.02 (0.01–0.03)	0.01	0.25	3.33	0.001		
Non-healthy behaviour	0.21 (0.04–0.39)	0.09	0.18	2.42	0.017		
Block 2							
Age	−0.03 (−0.06–−0.01)	0.01	−0.22	−2.94	0.004	0.18**	0.03*
Stress	0.02 (0.01–0.03)	0.01	0.25	3.32	0.001		
Non-healthy behaviour	0.20 (0.03–0.37)	0.09	0.17	2.27	0.024		
nonMHC-*d^2^*	−1.17 (−2.14–−0.20)	0.49	−0.18	−2.38	0.019^†^		
Block 3							
Age	−0.03 (0.05–−0.01)	0.01	−0.20	−2.59	0.011	0.20**	0.02*
Stress	0.02 (0.01–0.03)	0.01	0.24	3.27	0.001		
Non-healthy behaviour	0.20 (0.03–0.037)	0.09	0.17	2.33	0.021		
nonMHC-*d^2^*	−1.11 (−2.07–−0.15)	0.49	−0.17	−2.28	0.024		
MHC-*d^2^*	−0.98 (−1.95–−0.00)	0.49	−0.15	−1.98	0.049^‡^		

Covariates were entered in block 1 to investigate whether genetic diversity had an effect on health over and above the effect of the covariates. NonMHC-d^2^ was entered in block 2 to examine whether MHC-d^2^ had an effect on health over and above the effect of nonMHC-d^2^ (n = 153). See notes below for effect sizes and 95% confidence intervals.

Notes: Overall model: *F_5,147_* = 7.27, *p*<0.001.

**p<0.05, **p<0.001

Effect sizes: ^†^nonMHC-*d^2^*: *r* (95% CI) = −0.19 (−0.34–−0.04), ^‡^MHC-*d^2^*: *r* (95% CI) = −0.16 (−0.31–−0.003).

To test whether some or all of the nonMHC and MHC loci sampled contributed to the observed effects, we conducted single-locus analyses (see Lie et al. 2008), using consecutive hierarchical multiple regression models. As in the other models, we adjusted for gender, age, stress and non-healthy behaviours. In each model the predictors were *d^2^* at a single locus and a measure of *d^2^* calculated across all remaining loci, omitting the locus under consideration. No single locus disproportionally influenced the observed effect of nonMHC-*d^2^* on health, and the removal of any one locus did not substantially reduce the *p*-value for the effect of nonMHC-*d^2^* calculated across the remaining loci (Table 4).

**Table 4:** Test for the effect of single locus d^2^ and general d^2^ (calculated using all loci but excluding the locus being considered) effects on health (number of symptoms) shown separately for nonMHC and MHC loci (n = 153).

nonMHC-*d^2^*			MHC-*d^2^*		
Loci	Single locus *p*	General *p*	Loci	Single locus *p*	General *p*
D1S218	0.367	0.035	D6S276	0.508	0.192
D2S2382	0.567	0.031	D6S2863	0.710	0.133
D4S413	0.078	0.038	D6S510	0.598	0.116
D6S441	0.994	0.016	D6S265	0.164	0.028
D8S550	0.814	0.014	D6S2811	0.634	0.091
D10S191	0.150	0.080	D6S2810	0.782	0.130
D12S345	0.910	0.014	D6S2792	0.734	0.152
D14S283	0.058	0.071	D6S2787	0.497	0.133
D16S515	0.032	0.003	D6S2894	0.655	0.238
D18S61	0.584	0.024	D6S2883	0.005	0.608
D20S117	0.196	0.038	D6S2876	0.827	0.153
			D6S291	0.511	0.069

Table shows p-values for the single locus (single) and all remaining loci combined (general).

The effect of MHC-*d^2^* on health, however, was influenced by one locus in particular, D6S2883 (*p* = 0.005, Table 4), and the effect of the remaining loci combined was substantially reduced on its removal (*p*>0.60). However, the effect of this locus would not survive Bonferroni correction for multiple comparisons (corrected *p* = 0.004).

## Discussion

These results provide some support for an association between genetic diversity and a measure of general, everyday health in humans. We found a small, but significant, effect of nonMHC genetic diversity, measured as standardized mean-*d^2^*, on health. Individuals with greater nonMHC-*d^2^* reported significantly fewer symptoms over a four-month period than less diverse individuals, with nonMHC-*d^2^* accounting for 3% of the variance in health. This relationship suggests that the previously observed male preferences for the faces of females with high levels of nonMHC-*d^2^* would be adaptive for obtaining a healthier mate [46].

That nonMHC-*d^2^* is associated with health, a fitness-related trait, is consistent with several studies in non-human animals that report weak, positive relationships between genetic diversity (measured as mean *d^2^*) at neutral markers and fitness-related traits [7], [59], [61], [62], [73]. Although little is known about the effect of genetic diversity on disease resistance in humans, there is evidence that reduced genome-wide heterozygosity increases the risk of two severe infectious diseases, tuberculosis and hepatitis [9], and increases the incidence of a range of non-infectious diseases [10], [11]. Thus our results add to the evidence for a beneficial effect of genetic diversity on health in humans.

There was also a small effect of MHC-*d^2^* on health, with MHC-*d^2^* accounting for 2% of the variance in health. Unlike the effect of nonMHC-*d^2^*, this effect was mainly driven by allelic diversity (*d^2^*) at one locus, D6S2883. D6S2883 is in strong linkage with the MHC class II gene HLA-DRB1 [67], and heterozygosity at HLA-DRB1 has been implicated in resistance to both viral and parasite infections in humans [26], [27], [29]. However, the effect of the single locus did not survive correction for multiple comparisons, therefore leaving the statistical significance of this finding uncertain. Replication of this finding is clearly needed before any generalizations can be made. For example, future research could explore the role of Class II diversity, especially HLA-DRB1, in susceptibility to common infectious diseases such as upper-respiratory tract infections.

We found no effect of the heterozygosity measures on health. The discrepancy in the associations between the *H* and the *d^2^* measures and health may be a result of several factors. First, standardized mean *d^2^* has intrinsically higher variance and greater heterogeneity in effect sizes than *H*, especially when based on a small number of loci [1], which may explain some of the observed differences in associations with health.

Second, some researchers argue that heterozygosity is more sensitive to genetic diversity–fitness associations than mean *d^2^*, because heterozygosity tends to correlate more highly with known inbreeding coefficients than mean *d^2^* [57], [58], [70], [81]. By contrast, others have argued that under certain circumstances, mean *d^2^* measures can be more sensitive to diversity–fitness associations than heterozygosity, when highly variable markers are used in large outbred populations, and there is low levels of variation in heterozygosity but considerable variation in mean *d^2^* [60], [63], [82].

Here, heterozygosity levels were high across all individuals, and we used highly variable markers. Thus, increased allelic distance (captured by *d^2^*, but not *H*) may capture fitness benefits associated with health not explained by heterozygosity. For example, the *d^2^*-health relationship may reflect an outbreeding advantage, where greater allelic divergence predicts better health, rather than a selection against homozygotes [e.g. 61].

Lastly, although the two measures correlate positively (*r*>0.30), they appear to be sensitive to different aspects of phenotypic quality in humans (e.g. MHC-*H*, but not *d^2^*, predicts male facial attractiveness, while nonMHC-*d^2^*, but not *H*, predicts health and female attractiveness). Thus, more research is needed to better understand how and why *H* and *d^2^* predict fitness-related traits differently within the same individual.

Although we measured genetic diversity using microsatellites assumed to be selectively neutral, the MHC markers used here are known to be in linkage with MHC genes [67]. In addition, the nonMHC markers were highly polymorphic and therefore likely to be in linkage with functional loci under balancing selection [e.g. 68], [69]. Thus diversity at the microsatellites should reflect genetic diversity across the MHC and in general (nonMHC). The effects of nonMHC-*d^2^* on health did not appear to be unduly influenced by any one locus, while only one MHC locus contributed to the observed effect of MHC-*d^2^* on health.

From a sexual selection perspective, our finding that nonMHC-*d^2^* was associated with health provides some of the first evidence that genetic diversity outside the MHC is beneficial for the individual. This finding also suggests that male preferences for greater nonMHC-*d^2^* in females are adaptive. On the other hand, females prefer the faces of males who are MHC heterozygous [45], [46], but we found no association between MHC diversity and our measure of health. We cannot, however, conclude that the observed MHC heterozygosity preference is not adaptive because our health measure is by no means a comprehensive measure of mate quality, and there is good evidence that MHC heterozygosity is associated with greater resistance to diseases in humans [26]–[29].

In an attempt to capture individual differences in “everyday health”, our measure of health consisted of self-reported symptoms associated with common infectious diseases, such as upper-respiratory tract infections. Although a medical doctor did not verify the reported symptoms, Larson et al. [48] found that in 93 of 100 cases, the self-reported symptoms were confirmed by a doctor. They concluded that self-report of symptoms was an acceptable proxy measure of the presence of infectious diseases. Our health measure improved upon commonly used retrospective self-report measures [e.g. 30], [52], [53] by using a longitudinal design to reduce biases associated with self-report of symptoms. This design should improve reporting accuracy because the recall periods are short (two weeks only), and the participants are aware that they need to monitor and report their symptoms. In addition, we also controlled for variables that can impact on reporting of and susceptibility to infectious diseases such as negative affect and stress. Controlling for such covariates should reduce the variance in symptom reporting that is due to false alarms such as psychosomatic conditions without any infectious background.

Despite the relatively brief period surveyed (four months) and modest sample size, we still found significant effects of one measure of genetic diversity on “everyday health” in our student sample. Including an exam period, a stressful event known to compromise immunocompetence [54], [55], may have increased our ability to detect a genetic diversity-health relationship. Our effect sizes (*r* −0.16 and −0.19) compare favourably with those typically reported for heterozygosity-fitness associations (mean *r*>0.10) [1], and are consistent with the range of effect sizes normally reported in evolutionary and ecological research (*r*'s between 0.18–0.19) [83]. Thus, prospective health surveys, even over relatively short time periods, may be a promising avenue to further investigate relationships between genetic diversity and health in humans.

In conclusion, our results provide mixed evidence for an association between genetic diversity and “everyday health” in humans. Standardized mean *d^2^*, but not heterozygosity, at nonMHC loci predicted fewer symptoms reported over a four-month period. Previously, we found that the same measure of genetic diversity predicted facial attractiveness of the same females [46]. Combined, these findings suggest that this preference may be adaptive. Future research should investigate the relative merits of using standardized mean *d^2^* and heterozygosity as measures of genetic diversity when investigating the role of genetic diversity in natural and sexual selection acting on human populations.

## Supporting Information

Table S1 (0.08 MB DOC)

Table S2 (0.04 MB DOC)

Table S3 (0.05 MB DOC)
