# Supplementary material for: Does Genetic Diversity Predict Health in Humans?
Source: PLoS One. 2009 Jul 27;4(7):e6391. doi: 10.1371/journal.pone.0006391 (PMC2712076; doi:10.1371/journal.pone.0006391)
Supplement: Table S1 — (0.08 MB DOC) [file pone.0006391.s001.doc]

***Table S3.*** *The initial, full multiple regression model predicting health (number of symptoms) using nonMHC-d^2^ and MHC-d^2^*, including interaction-terms between gender and genetic diversity and adjusting for potential covariates (n = 153).

|  | B (SE) | *β* | *t* | p |
| --- | --- | --- | --- | --- |
| Gender | 0.003 (0.240) | 0.004 | 0.01 | 0.989 |
| Age | -0.027 (0.012) | -0.178 | -2.28 | 0.024 |
| SES | -0.023 (0.022) | -0.080 | -1.04 | 0.299 |
| Stress | 0.012 (0.006) | 0.188 | 1.98 | 0.050 |
| NA | 0.002 (0.002) | 0.075 | 0.83 | 0.409 |
| Non-healthy behaviour | 0.203 (0.089) | 0.174 | 2.28 | 0.024 |
| nonMHC-*d^2^* | -1.127 (0.492) | -0.172 | -2.29 | 0.024 |
| MHC-*d^2^* | -0.970 (0.499) | -0.148 | -1.94 | 0.054 |
| Gender*nonMHC-*d^2^* | 0.165 (0.494) | 0.066 | 0.33 | 0.739 |
| Gender * MHC-*d^2^* | -0.310 (0.492) | -0.132 | -0.63 | 0.530 |

Note. The full model was significant overall in predicting number of symptoms, F_10,142_ = 3.87, p < 0.001. When fitted without covariates, health was significantly predicted by both nonMHC- *d^2^* (B(SE) = -1.146 (0.516), *β =* -0.175, *p* = 0.028) and MHC- *d^2^* (B(SE) = -1.231 (0.518), *β =* -0.187, *p* = 0.019), overall model *F_2,150_* = 5.57, *p* =0.005, *R^2^* = 0.067.
