# Supplementary material for: Does Genetic Diversity Predict Health in Humans?
Source: PLoS One. 2009 Jul 27;4(7):e6391. doi: 10.1371/journal.pone.0006391 (PMC2712076; doi:10.1371/journal.pone.0006391)
Supplement: Table S2 — (0.04 MB DOC) [file pone.0006391.s002.doc]

*Table S1****.***Correlations between the various health (number of symptoms), covariates and genetic diversity variables, n = 153. Pearson product moment correlations are shown above the diagonal and Spearman’s rho correlations are shown below.

|  | Health | Gender | Age | SES | NA | Stress | N-H behav. | nonMHC-*H* | MHC-*H* | nonMHC-*d^2^* | MHC-*d^2^* |
| --- | --- | --- | --- | --- | --- | --- | --- | --- | --- | --- | --- |
| Health | 1 | -0.13 | -0.20** | -0.15 | 0.12 | 0.22** | 0.21** | 0.02 | 0.10 | -0.19* | -0.20* |
| Gender | -0.12 | 1 | 0.16 | 0.10 | -0.6 | -0.26** | 0.07 | -0.10 | -0.01 | -0.05 | 0.01 |
| Age | -0.22** | 0.23** | 1 | 0.08 | 0.14 | 0.10 | -0.05 | -0.15 | 0.10 | -0.07 | 0.16* |
| SES | -0.15 | 0.09 | 0.21** | 1 | -0.04 | -0.02 | -0.14 | -0.13 | 0.01 | 0.03 | 0.10 |
| NA | 0.12 | -0.06 | 0.17* | -0.03 | 1 | 0.61*** | 0.04 | -0.02 | -0.03 | 0.05 | 0.06 |
| Stress | 0.27** | -0.03** | 0.01 | -0.01 | 0.61** | 1 | 0.10 | -0.11 | 0.01 | -0.02 | -0.00 |
| N-H behav. | 0.22** | 0.07 | -0.02 | -0.10 | 0.07 | 0.12 | 1 | -0.05 | 0.04 | -0.07 | 0.00 |
| nonMHC-*H* | 0.00 | -0.10 | -0.17* | -0.10 | -0.05 | -0.14 | -0.07 | 1 | 0.011 | 0.34*** | -0.01 |
| MHC-*H* | 0.07 | 0.01 | 0.08 | 0.00 | -0.05 | -0.03 | 0.06 | 0.10 | 1 | -0.08 | 0.35*** |
| nonMHC-*d^2^* | -0.19* | -0.05 | -0.03 | 0.09 | 0.01 | -0.02 | -0.09 | 0.33** | -0.11 | 1 | 0.05 |
| MHC-*d^2^* | -0.18 | -0.00 | 0.13 | 0.11 | 0.05 | -0.01 | 0.03 | 0.03 | 0.37** | 0.03 | 1 |

Note. N-H behav. = non-healthy behaviours, *H* = heterozygosity, *d^2^* = standardized mean *d^2^*

* *p < 0.05*

*** p < 0.01*

****p < 0.001*
