# Supplementary material for: Does Genetic Diversity Predict Health in Humans?
Source: PLoS One. 2009 Jul 27;4(7):e6391. doi: 10.1371/journal.pone.0006391 (PMC2712076; doi:10.1371/journal.pone.0006391)
Supplement: Table S3 — (0.05 MB DOC) [file pone.0006391.s003.doc]

*Table S2*. *The initial, full multiple regression model predicting health (number of symptoms) using nonMHC-H and MHC-H, including interaction-terms between gender and genetic diversity and adjusting for potential covariates (n = 153).*

|  | B (SE) | *β* | *t* | p |
| --- | --- | --- | --- | --- |
| Gender | -0.592 (0.413) | -1.374 | -1.85 | 0.067 |
| Age | -0.030 (0.320) | -0.198 | -2.48 | 0.014 |
| SES | -0.023 (0.023) | -0.082 | -1.04 | 0.300 |
| Stress | 0.014 (0.006) | 0.205 | 2.11 | 0.036 |
| NA | 0.001 (0.002) | 0.047 | 0.48 | 0.620 |
| Non-healthy behaviour | 0.181 (0.091) | 0.155 | 1.99 | 0.049 |
| nonMHC-*H* | -0.078 (0.282) | -0.022 | -0.28 | 0.783 |
| MHC-*H* | 0.333 (0.273) | 0.096 | 1.22 | 0.224 |
| Gender*nonMHC-*H* | 0.424 (0.287) | 0.836 | 1.48 | 0.141 |
| Gender * MHC-*H* | -0.256 (0.273) | 0.505 | 0.94 | 0.350 |

Note. The full model was significant overall in predicting number of symptoms, *F_10, 142_* = 3.38, *p* = 0.001.
